# Scleral Thickness in Human Eyes

**DOI:** 10.1371/journal.pone.0029692

**Published:** 2012-01-06

**Authors:** Sujiv Vurgese, Songhomitra Panda-Jonas, Jost B. Jonas

**Affiliations:** Department of Ophthalmology, Medical Faculty Mannheim, Ruprecht-Karls-University Heidelberg, Heidelberg, Germany; Massachusetts Eye & Ear Infirmary, Harvard Medical School, United States of America

## Abstract

**Purpose:**

To obtain information about scleral thickness in different ocular regions and its associations.

**Methods:**

The histomorphometric study included 238 human globes which had been enucleated because of choroidal melanomas or due to secondary angle-closure glaucoma. Using light microscopy, anterior-posterior pupil-optic nerve sections were measured.

**Results:**

In the non-axially elongated group (axial length ≤26 mm), scleral thickness decreased from the limbus (0.50±0.11 mm) to the ora serrata (0.43±0.14 mm) and the equator (0.42±0.15 mm), and then increased to the midpoint between posterior pole and equator (0.65±0.15 mm) and to the posterior pole (0.94±0.18 mm), from where it decreased to the peri-optic nerve region (0.86±0.21 mm) and finally the peripapillary scleral flange (0.39±0.09 mm). Scleral thickness was significantly lower in the axially elongated group (axial length >26 mm) than in the non-axially elongated group for measurements taken at and posterior to the equator. Scleral thickness measurements of the posterior pole and of the peripapillary scleral flange were correlated with lamina cribrosa thickness measurements. Scleral thickness measurements at any location of examination were not significantly (all *P*>0.10) correlated with corneal thickness measurements. Scleral thickness was statistically independent of age, gender and presence of glaucoma.

**Conclusions:**

In non-axially elongated eyes, the sclera was thickest at the posterior pole, followed by the peri-optic nerve region, the midpoint between posterior pole and equator, the limbus, the ora serrata, the equator and finally the peripapillary scleral flange. In axially elongated eyes, scleral thinning occurred at and posterior to the equator, being more marked closer to the posterior pole and the longer the axial length was. Within the anterior and posterior segment respectively, scleral thickness measurements were correlated with each other. Posterior scleral thickness was correlated with lamina cribrosa thickness. Scleral thickness measurements at any location of examination were not significantly correlated with corneal thickness or with age, gender and presence of absolute secondary angler-closure glaucoma.

## Introduction

The sclera forms the outer layer of the ocular globe and serves to stabilize size and shape to the eye. Stability of the globe shape is of utmost importance for the optical system which heavily depends on constant distances between cornea, lens and retina. The thickness of the sclera is a major factor in providing a firm structure to the globe [1]–[5]. Although previous studies have already measured the scleral thickness in human eyes [6]–[25], these studies included relatively few globes, and quantitative information on differences in scleral thickness between various ocular regions and its association with the size of the globe, gender and lamina cribrosa thickness have been scarce so far. We, therefore, conducted this study to measure the scleral thickness in different regions of the eye and to correlate the measurements with data of the globe diameters, thickness of the lamina cribrosa and gender.

## Methods

### Ethics Statement

The Medical Ethics Committee II of the Medical Faculty Mannheim of the Ruprecht-Karls University Heidelberg approved the study protocol. In agreement with the approval by the ethics committee, informed consent was not obtained since the globes had been enucleated up to 30 years before the study was initiated.

The study included globes of white patients which had been enucleated due to painful absolute glaucoma or because of malignant choroidal melanomas. In the glaucomatous group, vision was completely or almost completely lost. The whole study population was divided into globes with an axial length longer than 26 mm (axially elongated group) and into globes with an axial length of equal to or less than 26 mm (non-axially elongated group). The reason to use a value of 26 mm as cut-off point between the axially elongated group and the non-axially elongated group was that in preceding clinical and population-based studies the equivalent of an axial length of about 26.0 to 26.5 mm marked the start of a high myopia associated increase in the optic nerve head size as well as an increase in the prevalence of myopic retinopathy [26]–[28]. In the tumor group, the malignant choroidal melanomas did not infiltrate the trabecular meshwork, neither directly or indirectly by migrating cells. The parapapillary region was free of tumor. Visual acuity depended on the degree of cataract, vitreous opacities, and foveal involvement by the tumor. At the time when the eyes were enucleated, no other treatment modalities such as endoresection of the tumor or radiologic brachytherapy were available or were thought not to be suitable for tumor removal with respect to its location and size. Some of the eyes had been included in previous studies on different topics [7], [8].

**Figure 1:**
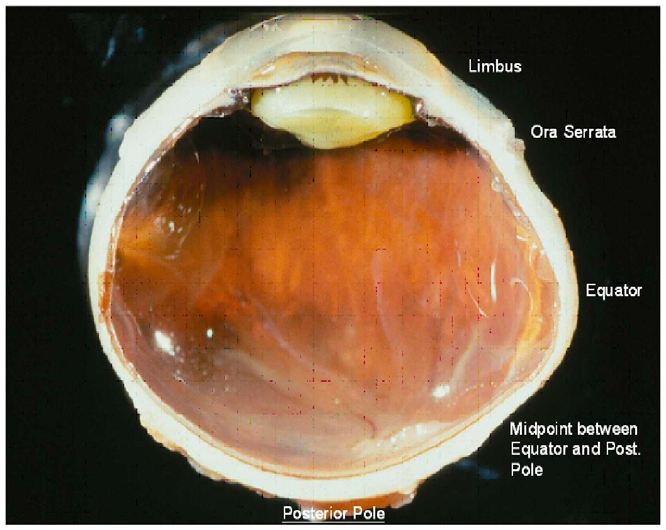
Transected human globe demonstrating the points of scleral measurements.

**Figure 2:**
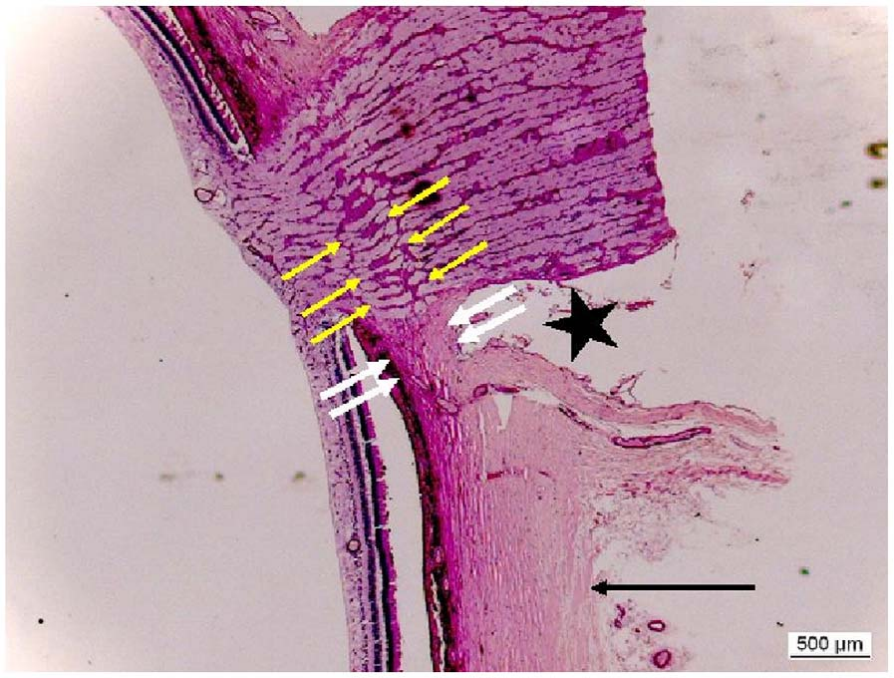
Histophotograph showing the peripapillary scleral flange (white double arrows) which functions as “bridge” between the sclera just outside of the optic nerve meninges (long black arrow) and the lamina cribrosa of the optic nerve head (yellow arrows), and which forms the anterior border of the orbital cerebrospinal fluid space (black asterisk).

Immediately after enucleation, the globes were fixed in a solution of 4% formaldehyde and 1% glutaraldehyde. Using anatomical landmarks such as the insertion of the oblique muscles, the 12 o'clock position of the globes was marked at the limbus. The axial length and the horizontal and vertical diameters of the globe were measured with a caliper. The globes were then processed for histological sectioning. The direction of the histologic cut depended on the location of the tumor in the group of eyes with a malignant choroidal melanoma, and it was horizontal in the glaucoma group. Otherwise the preparation of the globes did not vary between the glaucomatous eyes and the non-glaucomatous eyes or between the axially elongated and the non-axially elongated eyes. The globes were prepared in routine manner for light microscopy. A pupil-optic nerve section was cut out of the fixed globes. The segments were dehydrated in alcohol, imbedded in paraffin, sectioned for light microscopy, and stained by the Periodic-Acid-Shiff (PAS) method. For all eyes, one section (thickness: 8 µm) running through the central part of the optic disc was selected for further evaluation. We measured the thickness of the sclera at the:

limbus
ora serrata
equator
midpoint between the posterior pole and the equator
posterior pole (Fig. 1)
outside of the optic nerve head after merging of the optic nerve sheaths with the sclera (Fig. 2), and
at the border optic disc in the peripapillary scleral flange, before merging of the optic nerve sheaths with the sclera (Fig. 2).

Additionally, we measured the sagittal, horizontal and vertical diameter of the globe. In subgroups of eyes, we determined the thickness of the lamina cribrosa in the center of the lamina cribrosa, at the optic disc border, and at the intermediate locations between disc border and lamina cribrosa center, and we assessed the thickness of the cornea in the corneal center, at the limbus, and in the intermediate position between the corneal center and the limbus.

Statistical analysis was performed using SPSS for Windows, version 19.0 (IBM-SPSS, Chicago, Illinois, USA). In a first step, the distribution of the values was tested using the Kolmogorov-Smirnov-test. We found that the scleral thickness parameters did not show a Gaussian distribution. In a second step, we calculated the mean values ± standard deviations as well as the medians and the ranges of the measurements. In a third step of the statistical analysis, we compared the scleral thickness measurements between the various regions of the eye, using the non-parametric Wilcoxon-test for paired samples. In a fourth step of the statistical analysis, we compared the non-axially elongated group with the axially elongated group by applying the non-parametric Mann-Whitney-U-test for unpaired samples. Finally, we assessed potential associations between the scleral thickness measurements with age, gender and presence of glaucoma, first in a univariate analysis (calculating Pearson's correlation coefficient), and eventually in a multivariate analysis. The level of significance was 0.05 (two-sided) in all statistical tests.

## Results

The study included 238 human globes of 238 subjects with a mean age of 62.0±13.1 years (median: 62.0 years; range: 24–89 years). The whole study population was subdivided into 162 eyes enucleated due to a malignant choroidal melanoma, and 76 eyes enucleated because of secondary angle-closure glaucoma. Mean axial length was 25.2±3.1 mm (median: 24.0 mm; range: 20–39 mm). Sixty globes had an axial length of more than 26 mm and were defined to be axially elongated.

### Regional Differences

**Figure 3:**
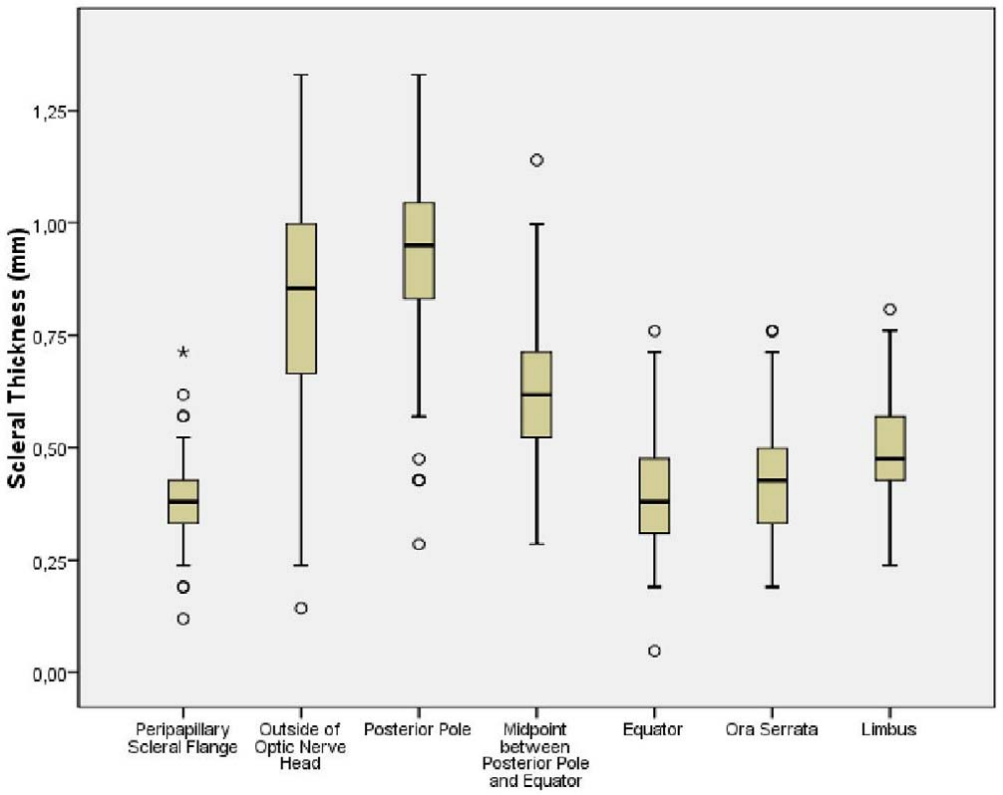
Scleral Thickness at Different Measurement Points in Human Enucleated Eyes.

In the total study population, scleral thickness decreased from the limbus to the ora serrata and the equator, and then increased to the midpoint between posterior pole and equator and to the posterior pole, from where it decreased to the peri-optic nerve region and finally the peripapillary scleral flange (Fig. 3) (Table 1). The sclera was significantly (*P*<0.001) thicker at the posterior pole than outside of the optic nerve after merging of the optic nerve sheaths with the sclera (peri-optic nerve region), where it was significantly (*P*<0.001) thicker than at the midpoint between posterior pole and equator, where it was significantly (*P*<0.001) thicker than at the limbus, where it was significantly (*P*<0.001) thicker than at the ora serrata, where it was significantly (*P*<0.001) thicker than at the equator, where it was significantly (*P* = 0.01) thicker than in the region of the peripapillary scleral flange (Table 1).

**Table 1:** Scleral Thickness Measurements (mm) (Mean ± Standard Deviation; Range) Obtained in Various Regions of Human Eyes with and without Absolute Secondary Angle-Closure Glaucoma.

	Total Group	Non-Axially Elongated	Axially Elongated	*P*-Value
Sclera Thickness at		Group	Group	
Posterior pole	0.86±0.26 (0.05, 1.52)	0.94±0.18 (0.43, 1.33)	0.67±0.33 (0.10, 1.23)	<0.001
Outside of optic nerve head (merged	0.79±0.25 (0.10, 1.33)	0.86±0.21 (0.24, 1.33)	0.63±0.28 (0.14, 1.23)	<0.001
optic nerve sheaths with sclera)				
Midpoint between posterior	0.59±0.19 (0.10, 1.14)	0.65±0.15 (0.33, 1.14)	0.47±0.19 (0.10, 1.00)	<0.001
pole and equator				
Limbus	0.50±0.10 (0.24, 0.85)	0.50±0.11 (0.24, 0.85)	0.46±0.06 (0.38, 0.62)	0.17
Ora serrata	0.42±0.13 (0.14, 0.95)	0.43±0.14 (0.14, 0.95)	0.40±0.13 (0.19, 0.76)	0.32
Equator	0.40±0.14 (0.05, 0.85)	0.42±0.15 (0.15, 0.85)	0.35±0.14 (0.14, 0.76)	0.002
Peripapillary scleral flange	0.37±0.11 (0.10, 0.76)	0.39±0.09 (0.14, 0.76)	0.33±0.12 (0.10, 0.66)	0.003
Lamina cribrosa thickness (µm)				
Center	259±232 (19, 1353)	321±256 (19, 1353)	108±93 (26 , 313)	0.006
Midperipheral	247±208 (20, 833)	298±214 (20, 833)	114±102 (31, 400)	0.014
Optic Disc Border	258±224 (18, 1149)	311±237 (18, 1149)	116±107 (28, 422)	0.01

*P*-Value: Statistical significance of the difference between the axially elongated group and the non-axially elongated group (Mann-Whitney-U-test for unpaired samples).

Axial elongation was defined as an axial length of the fixated human globes of >26 mm.

In a similar manner in the non-axially elongated group, the sclera was significantly (*P*<0.001) thicker at the posterior pole than outside of the optic nerve after merging of the optic nerve sheaths with the sclera, where it was significantly (*P*<0.001) thicker than at the midpoint between posterior pole and equator, where it was significantly (*P*<0.001) thicker than at the limbus, where it was significantly (*P*<0.001) thicker than at the ora serrata, where it was slightly (*P* = 0.058) thicker than at the equator, where it was slightly (*P* = 0.076) thicker than in the region of the peripapillary scleral flange (Table 1).

Since the scleral thickness measurements obtained at and posterior to the equator were significantly thinner in the axially elongated group than in the non-axially elongated group, the inter-regional differences in scleral thickness between the posterior pole and the anterior segment (limbus and at the ora serrata) were less marked in the axially elongated group than in the non-axially elongated group (Table 1). In the axially elongated group, the sclera was still significantly (*P* = 0.012) thicker at the posterior pole than outside of the optic nerve after merging of the optic nerve sheaths with the sclera, where it was significantly (*P*<0.001) thicker than at the midpoint between the posterior pole and the equator. The sclera thickness measurements obtained at the midpoint between posterior pole and equator and at the limbus did not differ significantly (*P* = 0.51), nor did the measurements taken at the limbus and at the ora serrata (*P* = 0.21). At the ora serrata, the sclera was significantly thicker (*P* = 0.007) than at the equator. The measurements at the equator and the peripapillary scleral flange did not vary significantly in the axially elongated group (*P* = 0.32) (Table 1).

### Differences axially elongated eyes versus non-axially elongated eyes

**Figure 4:**
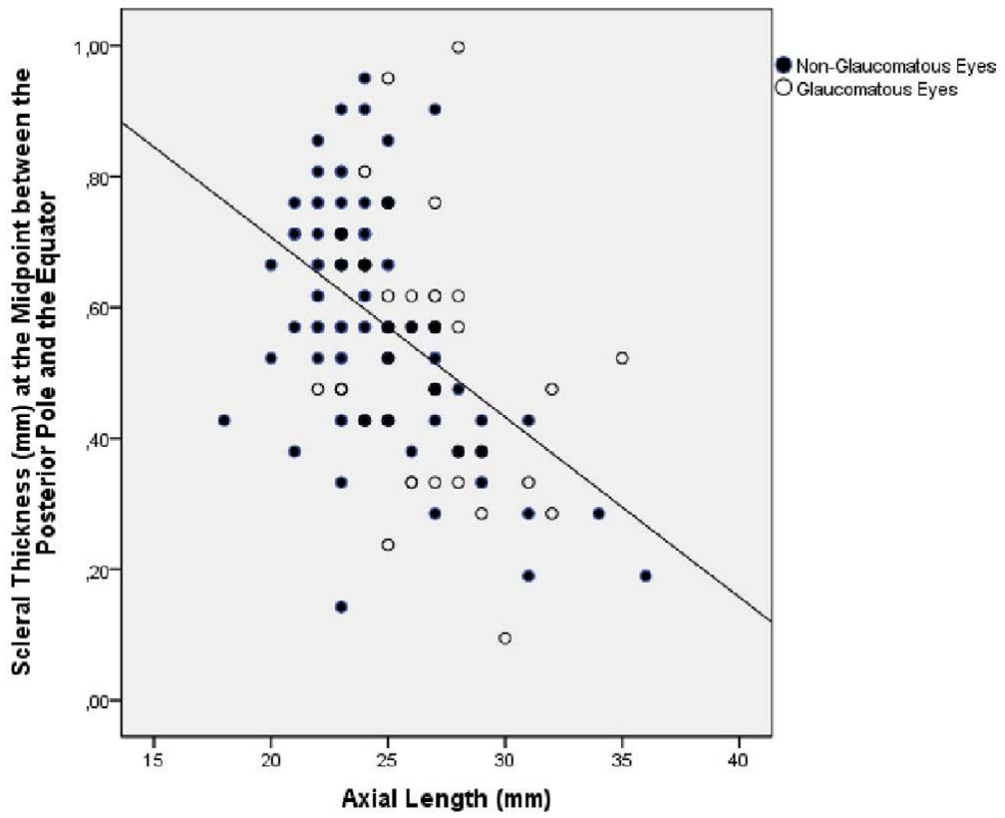
Scatterplot showing the correlation between axial length and the scleral thickness measured at the midpoint between the posterior pole and the equator in human globes (correlation coefficient = 0.48; *P*<0.001). Blue Circles The association was not statistically significant (*P* = 0.23), if only non-axially elongated eyes (axial length ≤26 mm) were included.

**Figure 5:**
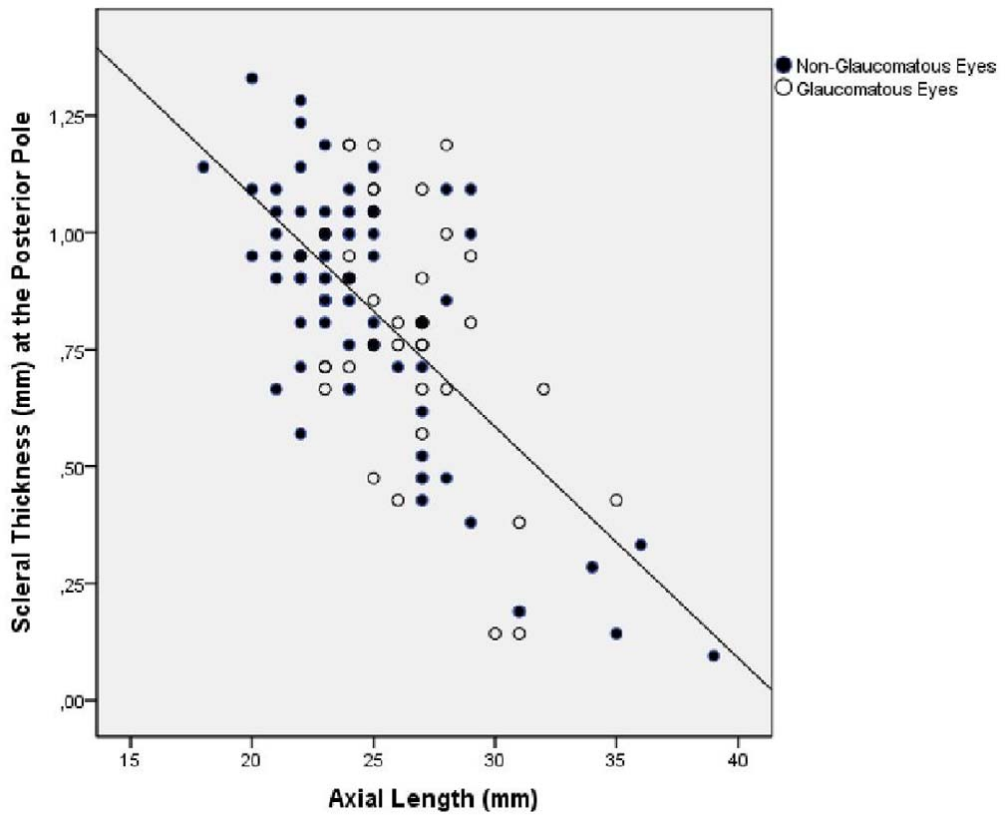
Scatterplot showing the correlation between axial length and the scleral thickness measured at the posterior pole in human globes (correlation coefficient = 0.61; *P*<0.001).

Comparing the axially elongated group with the non-axially elongated group revealed that the scleral thickness measurements were significantly lower in the axially elongated group for the measurement locations at and posterior to the equator (Table 1). The differences were more marked the closer to the posterior pole the measurements were obtained. Correspondingly, the scleral thickness measurements taken posterior to the equator were significantly correlated with axial length (Fig. 4, 5). The correlation coefficients were higher and the *P*-values lower, the closer the measurements were located to the posterior region (Table 2). If non-axially elongated eyes only were included into the statistical analysis, the associations between scleral thickness measurements and axial length were not statistically significant except for the association between axial length and the scleral thickness measured at the posterior pole (Table 3).

**Table 2:** Correlations between axial length and scleral thickness measurements obtained in different regions of the eye (whole study population).

Scleral Thickness at:	*P*-Value	Correlation	Regression	95% Confidence
		Coefficient	Coefficient	Interval
Limbus	0.51			
Ora serrata	0.28			
Equator	0.001	0.23	−0.010	−0.016, −0.004
Midpoint between equator	<0.001	0.50	−0.031	−0.038, −0.024
and posterior pole				
Posterior pole	<0.001	0.62	−0.052	−0.061, −0.043
Peri-optic nerve	<0.001	0.51	−0.045	−0.056, −0.035
Peripapillary scleral flange	<0.001	0.30	−0.013	−0.019, −0.007

**Table 3:** Correlations between axial length and scleral thickness measurements obtained in different regions of the eye in non-axially elongated eyes.

Scleral Thickness at	*P*-Value	Correlation	Regression	95% Confidence
		Coefficient	Coefficient	Interval
Limbus	0.95	0.01	−0.001	−0.021, 0.020
Ora serrata	0.11	0.14	−0.017	−0.039, 0.004
Equator	0.07	0.14	−0.375	−0.787, 0.037
Midpoint between equator	0.23	0.10	−0.012	−0.033, 0.008
and posterior pole				
Posterior pole	0.001	0.26	−0.039	−0.062, −0.016
Peri-Optic nerve	0.12	0.13	−0.023	−0.051, 0.006
Peripapillary scleral flange	0.53	0.05	−0.004	−0.018, 0.009

### Associations between scleral thickness measurements

Taking the whole study population, scleral thickness measurements from the anterior ocular segment were generally correlated with each other and they were not significantly correlated with measurements from the posterior segment. In a similar manner, posterior scleral thickness measurements were correlated with each other, however, they were not correlated with anterior scleral thickness measurements (Table 4). The same held true if the whole study population was divided into the non-axially elongated subgroup (Table 5) and the axially elongated subgroup (Table 6).

**Table 4:** Table showing the correlations (*P*-values and correlation coefficients) between scleral thickness measurements obtained in different ocular regions in all eyes of the study population.

	Limbus	Ora serrata	Equator	Midpoint	Peri Optic Nerve	Peripapillary Scleral Flange	Posterior Pole
Limbus	---	<0.001; 0.37	0.08	0.11	0.03; 0.20	0.85	0.59
Ora serrata		----	<0.001; 0.54	0.52	0.37	0.47	0.88
Equator			----	<0.001; 0.31	0.02; 0.16	0.006; 0.20	<0.001;0.25
Midpoint				----	<0.001; 0.66;	<0.001; 0.33	<0.001; 0.63
Peri Optic Nerve					----	<0.001; 0.44	<0.001; 0.83
Peripapillary Scleral Flange						----	<0.001; 0.48

**Table 5:** Table showing the correlations (*P*-values and correlation coefficients) between scleral thickness measurements obtained in different ocular regions in non-axially elongated human eyes.

	Limbus	Ora serrata	Equator	Midpoint	Peri Optic Nerve	Peripapillary Scleral Flange	Posterior Pole
Limbus	---	<0.001; 0.41	0.09	0.048;0.20	0.04; 0.21	0.58	0.32
Ora serrata		-----	<0.001; 0.56	0.06	0.94	0.09	0.12
Equator			----	<0.001; 0.31	0.06; 0.16	0.04; 0.18	<0.001;0.29
Midpoint				----	<0.001; 0.53;	0.05; 0.17	<0.001; 0.49
Peri Optic Nerve					----	0.002; 0.27	<0.001; 0.74
Peripapillary Scleral Flange						----	<0.001; 0.36

**Table 6:** Table showing the correlations (*P*-values and correlation coefficients) between scleral thickness measurements obtained in different ocular regions in axially elongated human eyes.

	Limbus	Ora serrata	Equator	Midpoint	Peri Optic Nerve	Peripapillary Scleral Flange	Posterior Pole
Limbus	---	0.85	0.47	0.35	0.09	0.06	0.12
Ora serrata		-----	<0.001; 0.53	0.37	0.18	0.50	0.21
Equator			----	0.23	0.90	0.18	0.91
Midpoint				----	<0.001; 0.71;	0.001; 0.52	<0.001; 0.60
Peri Optic Nerve					----	<0.001; 0.68	<0.001; 0.86
Peripapillary Scleral Flange						----	<0.001; 0.65

### Associations with lamina cribrosa thickness

**Figure 6:**
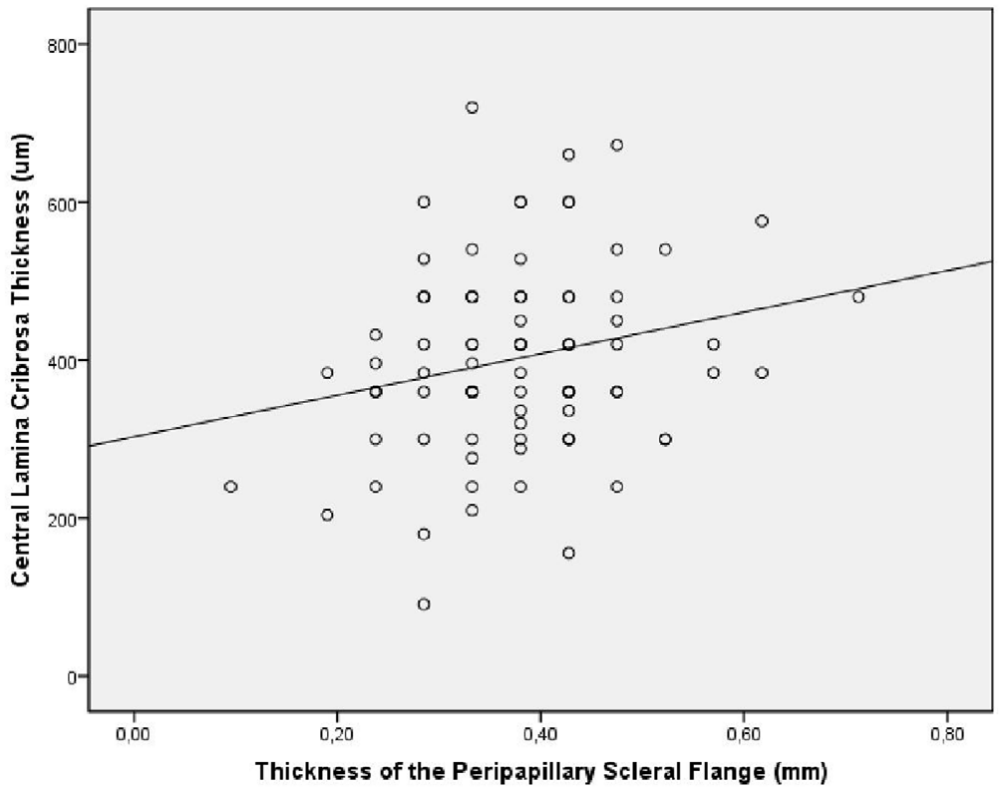
Scatterplot showing the correlation between the thickness of the peripapillary scleral flange and the central lamina cribrosa thickness in adult human non-glaucomatous eyes (*P* = 0.03; correlation coefficient: 0.23); equation of the regression line: Central Lamina Cribrosa Thickness (um) = 263×(Thickness of the Peripapillary Scleral Flange (mm))+303 um.

When we searched for an association between the scleral thickness measurements and the lamina cribrosa thickness, the statistical analysis included only eyes, for which lamina cribrosa thickness measurements were available and which did not have glaucoma (n = 89 eyes), since glaucoma is associated with a thinning of the lamina cribrosa [7]. It revealed that the central lamina cribrosa thickness was significantly correlated with the thickness of the peripapillary scleral flange (*P* = 0.03; correlation coefficient r = 0.23) (Fig. 6), of the sclera just outside of the optic nerve (*P* = 0.002; r = 0.31), and the sclera at the posterior pole (*P* = 0∶03; R = 0.23).

### Associations with corneal thickness

For the assessment of a potential association between the sclera thickness measurements and the thickness data of the cornea, we included eyes, for which corneal thickness were available (n = 122 eyes). It revealed that none of the corneal thickness parameters (central, intermediate position, limbus) was significantly (all *P*>0.10) correlated with any of the scleral measurements at the various locations of the globe. If the whole study population was differentiated into axially elongated eyes versus non-axially elongated eyes, the scleral thickness measurements in both groups at any location of examination were not significantly (all *P*>0.10) associated with any corneal thickness measurement [10].

### Associations with age, gender and presence of glaucoma

In univariate analysis, none of the scleral thickness measurements were significantly associated with age (all *P*>0.20), gender (all *P*>0.15), and presence of glaucoma (all *P*>0.10). In multivariate analysis with the scleral thickness parameter as dependent parameter and age, gender and presence of glaucoma as independent variables, all scleral thickness parameters were not significantly with any of these three variables (all *P*>0.10).

## Discussion

In the non-axially elongated eyes of our histomorphometric study, the sclera was thickest at the posterior pole, followed by the peri-optic nerve region, the midpoint between posterior pole and equator, the limbus, the ora serrata, the equator and finally the peripapillary scleral flange. In the axially elongated group, scleral thinning occurred at and posterior to the equator. The scleral thinning associated with axial elongation was more marked closer to the posterior pole and it was correlated with axial length. In the non-axially elongated eyes, scleral thickness was not significantly associated with axial length except for the scleral thickness at the posterior pole. For all eyes included into the study, scleral thickness measurements from the anterior ocular segment were correlated with each other, and scleral thickness measurements from the posterior segment were associated with each other. There were no associations in scleral thickness between the anterior segment and posterior segment (except of a modestly significant (*P* = 0.04) association between limbal scleral thickness peripapillary scleral thickness). In non-glaucomatous eyes, scleral thickness measurements of the posterior pole including of the peripapillary scleral flange were correlated with lamina cribrosa thickness measurements. The cut-off point for axial elongation with a posterior scleral thinning was in the present study at an axial length of approximately 26 mm of fixed human globes. Scleral thickness was statistically independent of age, gender and presence of glaucoma.

The scleral thickness measurements obtained in our study agreed with those reported in previous studies. In the study by Olsen and colleagues on 55 formalin-fixed human eye bank eyes, mean scleral thickness was 0.53±0.14 mm at the corneoscleral limbus (in our study: 0.50±0.10 mm), it decreased significantly to 0.39±0.17 mm near the equator (in our study: 0.40±0.14 mm), and increased 0.9 to 1.0 mm near the optic nerve (in our study: 0.86±0.26 mm) [6]. In a recent study by Norman and colleagues on 11 enucleated human globes, high-field micro magnetic resonance imaging revealed a mean thickness over the whole sclera of 0.67±0.80 mm (range: 0.56–0.83 mm) [20]. The maximum thickness was found at the posterior pole with a mean scleral thickness of 1.0±0.18 mm (in our study: 0.86±0.26 mm). The scleral thickness was lower at the equator (0.49±0.09 mm) (in our study: 0.40±0.14 mm). The lower measurements in our study may be explained by tissue shrinkage caused by the histological processing. The thickness of the peripapillary scleral flange had previously been measured in another investigation on a similar set of human globes by another primary examiner (JBJ), with similar results as obtained in the present study (non- axially elongated group: 0.38±0.06 mm versus 0.39±0.09 mm (this study); axially elongated group: 0.30±0.14 mm versus 0.33±0.12 mm (this study)).

The scleral thickness measurements from the anterior ocular segment were correlated with each other, and the scleral thickness measurements from the posterior ocular segment were correlated with each other. The scleral thickness measurements from the anterior ocular segment, however, were not correlated with the scleral thickness measurements from the posterior ocular segment. The reason for this finding may be that a myopia associated elongation of the globe, even in moderate myopia, may primarily take place in the posterior segment, so that any correlation between anterior and posterior scleral thickness measurements is confounded by the myopia associated elongation of the posterior ocular segment. Correspondingly, if a multivariate analysis was performed with anterior scleral thickness (e.g. measured at the ora serrata) as dependent variable and scleral thickness at the posterior pole and axial length as independent variables, anterior scleral thickness was significantly (*P* = 0.03) associated with posterior with posterior scleral thickness.

The differences in scleral thickness between the various ocular regions and the sequence of the ocular regions with respect to the scleral thickness have not been described in detail before. Interestingly, the thinnest part of the sclera was located in the peripapillary scleral flange, which made out about 50% of the scleral thickness in the peri-optic nerve region (just after merging of the optic nerve meninges with the sclera) and they made out less than 50% of the scleral thickness at the posterior pole (Table 1) (Fig. 2). It suggests that a region of least resistance (“locus minoris resistenciae”) was located in the region of the peripapillary scleral flange. Correspondingly, a traumatic avulsion of the optic nerve occurs at the peripapillary scleral flange with a rupture of the peripapillary scleral flange and the retinal nerve fibers leading to a retraction of the proximal part of the optic nerve and leaving the optic nerve meninges forming a hollow tube behind the eye. Considering that the peripapillary scleral flange is the thinnest part of the whole sclera and taking into account the ocular pulse related fluctuations in intraocular pressure, one may think that the whole optic nerve head may perform sagittal pulse related movements with the peripapillary scleral flange acting as hinge. This potential function of the peripapillary scleral flange may be possible since the peripapillary scleral flange is the only part of the sclera which borders the cerebrospinal fluid space and which does not border the retrobulbar orbital adipose tissue. The relationship between the thickness of the peripapillary scleral flange and scleral thickness in other ocular regions may also be interesting since the lamina cribrosa anchors in the peripapillary scleral flange, so that the lamina cribrosa may anatomically be considered as an elongation of the peripapillary scleral flange. Correspondingly, the thickness of the peripapillary scleral flange was correlated with the thickness of the lamina cribrosa (Fig. 6) in the non-glaucomatous eyes.

The thickness measurements of the sclera in the region of the equator are of importance for scleral buckling surgery, since the encircling bands are usually fixated in the region of the equator. The risk of an unintentional scleral perforation with the needle increases with decreasing thickness of the sclera. The lowest scleral thickness in the equator region in the axially elongated group was 0.14 mm (Table 1).

Interestingly, the scleral thickness was not significantly correlated with age (all *P*>0.20), gender (all *P*>0.15), and presence of glaucoma (all *P*>0.10). It indicates that in adults, scleral thickness does not markedly change during lifetime. It also indicates and confirms previous studies [29], that absolute secondary angle-closure glaucoma does not lead to pronounced changes in the thickness of the peripapillary scleral flange or any other part of the sclera. This finding is in contrast to investigations in monkeys which developed a posterior scleral thinning in the course of experimental high-pressure glaucoma [30].

Potential limitations of our study should be mentioned. First, due to postmortem swelling of the tissue after enucleation and due to the histological preparation of the slides, the measurements given in this study will not represent dimensions as determined in vivo. It was, however, not the purpose of the present investigation to evaluate the thickness of the sclera in real dimensions, but to compare the scleral thickness measurements between various regions of the eye. The systemic error which was introduced by the histological preparation of the slides affected the specimen of various eye regions presumably in a similar manner. Second, the study did not include normal human eyes but eyes which were enucleated either due to a malignant choroidal melanoma or due to end-stage glaucoma. It is, therefore, not clear whether the results of our study can be generally transferred onto normal human eyes. The statistical analysis showed, however, that the presence of glaucoma was not significantly associated with scleral thickness. Generally, however, one cannot exclude some degree of glaucoma-related expansion of the sclera, particularly since some of the patients were relatively young. Third, serial sections of the globes were not available so that it was not possible to determine, whether the histological section was located in the very center of the optic disc or whether it ran slightly paracentrally. This limitation held true, however, for the axially elongated group and for the non-axially elongated group. Fourth, the histological sections of the melanoma group were orientated according to the main location of the tumor, while the glaucomatous globes had been opened in a horizontal direction. The strength of our study is that it included human eyes which physiologically differ from monkey eyes in the thickness of the lamina cribrosa, sclera and other ocular tissues, and potentially in biomechanical properties. In addition, the number of axially elongated eyes in our study sample was relatively high.

In conclusion, in non-axially elongated eyes, the sclera was thickest at the posterior pole, followed by the peri-optic nerve region, the midpoint between posterior pole and equator, the limbus, the ora serrata, the equator and finally the peripapillary scleral flange. In axially elongated eyes, scleral thinning occurred at and posterior to the equator, being more marked closer to the posterior pole and the longer the axial length was. Within the anterior and posterior segment respectively, scleral thickness measurements were correlated with each other. Posterior scleral thickness was correlated with lamina cribrosa thickness. Scleral thickness measurements at any point of examination were not significantly correlated with corneal thickness or with age, gender and presence of absolute secondary angler-closure glaucoma.
